# Nomograms to predict the long‐time prognosis in patients with alpha‐fetoprotein negative hepatocellular carcinoma following radical resection

**DOI:** 10.1002/cam4.2944

**Published:** 2020-02-25

**Authors:** Jian Huang, Fu‐Chen Liu, Li Li, Wei‐Ping Zhou, Bei‐Ge Jiang, Ze‐Ya Pan

**Affiliations:** ^1^ Third Department of Hepatic Surgery Eastern Hepatobiliary Surgery Hospital Second Military Medical University Shanghai China; ^2^ Department of Nephrology Eastern Hepatobiliary Surgery Hospital Second Military Medical University Shanghai China

**Keywords:** alpha‐fetoprotein, carcinoma, hepatectomy, hepatocellular, nomogram

## Abstract

**Background:**

To develop and validate nomograms that can be used to predict outcomes in individuals suffering alpha‐fetoprotein (AFP) negative hepatocellular carcinoma (HCC) after radical resection.

**Methods:**

A total of 509 AFP‐negative HCC patients who received hepatectomy between January 2009 and March 2013 in our center were randomized into training and validation cohorts. Nomograms for both overall and recurrence‐free survival (OS and RFS, respectively) were established based on the predictors in the training cohort. Nomograms performance and discriminative power were assessed with concordance index (C‐index) values and decision curve analyses (DCA). The results were validated in the validation cohort.

**Results:**

Alkaline phosphatase, liver cirrhosis, tumor size, satellite lesions, microvascular invasion, and Edmondson‐Steiner grade were significantly linked to OS and RFS. Sex and tumor number were additional predictors for RFS. The OS nomogram had a C‐index value of 0.742, which was better than that for the AJCC eighth edition (0.632), BCLC system (0.553), and JIS score (0.557) (all *P* < .001). The RFS nomogram C‐index was 0.669, which was also superior to that of the AJCC eighth (0.608), BCLC stage (0.554), JIS score (0.551), and model of Gan et al (0.636) (*P* < .05 for all). Calibration curves indicated a good agreement between observed actual outcomes and predicted values. Kaplan‐Meier curves and DCA indicated that nomograms were powerful in discrimination and clinical usefulness. These results were supported by the validation cohort.

**Conclusions:**

These nomograms presented more accurate prognostic prediction in patients with AFP‐negative HCC after hepatectomy.

## Graphical abstract

About 30% of HCC patients showed negative serum AFP and had special clinicopathologic characteristics and prognosis. We developed and validated nomograms for predicting prognosis of patients with AFP‐negative HCC after radical resection. Our novel nomograms presented superior performance and discrimination ability than conventional staging systems based on the C‐indexes, calibration curves, decision curve analysis and Kaplan‐Meier curves, which were proposed for clinical decision.

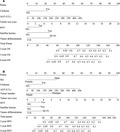

## 1 INTRODUCTION

Hepatocellular carcinoma (HCC) remains the third most common cause of cancer‐associated death.^1^ Approximately 80% of cases of HCC are linked to hepatitis B virus (HBV) infection and aflatoxin B1 exposure.^2^ China is among the worst‐hit countries for HCC. Liver transplantation and surgical resection are the appropriate treatment options for HCC patients, except those with advanced HCC.^1^ However, the long‐time prognosis for HCC after curative resection remains disappointing because of the high rate of tumor recurrence, reported to be 70% of cases after 5 years.^3^ Fortunately, surveillance programs, individualized treatment and careful follow‐up may improve the prognosis for patients with HCC.

Alpha‐fetoprotein (AFP) is a glycoprotein synthesized by fetal hepatocytes in addition to yolk sac cells.^4^ First described as a biomarker for HCC in the 1960s,^5^ it is often used in the diagnosis of HCC. Nevertheless, AFP sensitivity in diagnosing HCC is only 60% at the most efficient cut‐off value (10‐20 ng/mL) with low specificity,^6^ owing to serum AFP being also positive in cirrhosis, hepatitis and other malignancies.^7^ Thus, the use of serum AFP for the accurate diagnosis of HCC is controversial and confused. It was reported that only 60%‐70% of patients with HCC had observed elevated serum AFP, and up to 30% of HCC patients showed negative serum AFP.^8^ HCC patients with negative serum AFP have special clinicopathologic characteristics and prognosis, thus, a reliable model to accurately predict prognosis of AFP‐negative HCC may be of value for guiding clinical decision making.

Many staging systems have been employed to predict how HCC patients will respond over time, such as the AJCC eighth edition,^9^ JIS score,^10^ Okuda score,^11^ Cancer of the Liver Italian Program,^12^ and BCLC.^13^ However, these systems with different staging criteria had controversial clinical outcomes, even when patients in a given stage have received the same treatment. Furthermore, these systems are not specifically designed to predict outcomes of HCC when patients are AFP‐negative. Recently, nomograms constructed by variables are commonly performed in many cancer types,^14^, ^15^ some researcher proposing that nomograms could be regarded as a new prognostic standard.^16^ Nomograms for prediction of survival and recurrence of HCC in AFP‐negative patients after radical resection have been developed in two previous studies,^17^, ^18^ respectively. However, HCC patients with advanced stage who were not recommended for curative resection were included in both studies.

The present study aims to establish nomograms for survival and recurrence in HCC patients with AFP‐negative following radical resection. In addition, a comparison between the constructed nomograms and traditional staging systems was conducted to determine whether the nomograms provided more accurate prediction in prognosis.

## 2 MATERIALS AND METHODS

### 2.1 Patients

HCC patients who had negative serum AFP results when assessed preoperatively and who had bene treated via radical resection between January 2009 and March 2013 at the Eastern Hepatobiliary Surgery Hospital were retrospectively reviewed. Included patients met the following criteria: (a) an HCC diagnosis confirmed via pathology; (b) preoperative serum AFP < 20 μg/L; (c) Child‐Pugh A or B liver function; (d) initially treated by curative resection and no history of preoperative treatment. Excluded patients were those with: (a) other malignancies; (b) extrahepatic metastasis and lymph node metastasis; (c) macrovascular invasion such as portal veins, hepatic veins and inferior cava vein; (d) incomplete clinical or follow‐up data. The Clinical Research Ethics Committee of Eastern Hepatobiliary Surgery Hospital approved this study, with patients giving informed consent before surgery.

### 2.2 Preoperative management and surgery

Routine preoperative laboratory tests included liver function tests, hepatitis B and C virus detection, HBV deoxyribonucleic acid (HBV‐DNA) load, AFP, carcinoembryonic antigen (CEA), and carbohydrate antigen 19‐9 (CA19‐9). Abdominal b‐ultrasound and chest radiography were conducted routinely. Contrast‐enhanced computed tomography (CT) and/or magnetic resonance imaging (MRI) were used for assessing tumor status and extent of surgery, and three‐dimensional CT images were performed if necessary.

Hepatectomy was considered when patients were in good general condition, all tumor nodules could be resected and the residual liver volume was sufficiency. Anatomic resection was the preferred method for tumors distributed in a segment, lobe or hemi liver. Clamp‐crushing technique was performed for liver parenchyma separation. And Pringle's maneuver was operated for hepatic portal occlusion if needed. Major resection was defined as three or more Couinaud liver segments were resected.^19^ All included patients were received curative resection, as determined based on a lack of residual tumor tissues as well as a negative microscopic surgical margin.^20^

### 2.3 Definitions

The cut‐off value of HBV‐DNA load was defined as depicted in a previous paper.^21^ Microvascular invasion(MVI) was determined based upon tumor cell nests present in small branches of hepatic and portal veins and lymphatic ducts on microscopy.^22^ Satellite nodules were defined as separate lesions with similarly histological characteristics to the primary tumor within 2 cm both in size and distance.^23^ Tumor differentiation grade was determined according to the Edmondson‐Steiner classification.^24^

### 2.4 Follow‐up

Follow‐up was conducted every 3 months in the first year after hepatectomy and every 6 months subsequently. The routine examination included tumor markers, liver function, and abdominal b‐ultrasound. Abdominal CT and/or MRI was conducted every 6 months or when serum AFP continuous elevation. Diagnostic criteria for recurrence: newly detected lesions based on two or more imaging studies. Overall and recurrence‐free survival (OS and RFS, respectively) were study endpoints, with the former being the time from surgery to death or most recent follow‐up, and the latter being the time from surgery to first diagnosis of recurrence. All patients were followed up until March 2016.

### 2.5 Statistical analysis

SPSS v23 (IBM Corp.) and Prism v6.0 (GraphPad Software) were used for statistical testing. Continuous variables with abnormal distribution were medians (range), with Mann‐Whitney U tests used for comparing groups of data. Categorical data are described as the count (percentage) and chi‐squared or Fisher's exact test were used for comparisons. Survival was compared via the Kaplan‐Meier method and log‐rank test. A reverse Kaplan‐Meier method was used to calculate median follow‐up time. Factors independently associated with OS and RFS were identified via univariate and Cox forward stepwise regression analysis. The multivariate analysis results in the training cohort were used to generate nomograms with the package of *rms* in R version 3.5.1 (http://www.r-project.org/). Nomogram prediction accuracy was quantified by the concordance index (C‐index). The difference of C‐index between nomograms and other staging systems were compared via rcorrp.cens in *Hmisc* in R.^15^ Consistency between actual patient outcomes and predicted outcomes was assessed using calibration curves via the Kaplan‐Meier method. The C‐index and calibration curves of validation cohort were carried out in the same methods. Decision curve analysis (DCA) based on the net benefit was also depicted by the package of *rmda* in R.^25^ Three groups of low, middle and high risk of prognosis were divided by the cut‐off value based on the total points generated from the established nomogram by using the X‐tile software.^26^ *P* < .05 in two‐tailed was the significance threshold.

## 3 RESULTS

### 3.1 Basic clinicopathologic characteristics

In total, 509 patients were enrolled in this study, being separated at random into a training cohort (n = 339) and a validation cohort (n = 170) in the ration of 2:1 by the method of random number table. Clinicopathologic characteristics of individuals in the training and validation cohorts are given in Table 1, with the only significant difference between cohorts being in serum CEA levels. Most patients were male (89.4%), 89.1% of patients were HBsAg positive, 41.9% of patients had HBV‐DNA load more than 2000 IU/mL, and liver cirrhosis were identified in 171(50.4%) cases in the training cohort. In addition, almost all cases had a good liver function, single nodule was detected in 311 (91.7%) patients, the median diameter of tumor size was 4.1 (range 1.0‐20.7) cm, and 75 (22.1%) patients received major resection. The degree of tumor differentiation in 236 (69.6%) patients were Edmondson‐Steiner grade III + IV. MVI and satellite lesions positive were verified in 73 (21.5%) and 62 (18.4%) cases, respectively. Most patients were classified as AJCC eighth stage IB (63.7%), BCLC stage A (83.8%) and JIS score 1 (83.8%) in the training cohort.

**Table 1:** Clinicopathologic characteristics of all included HCC patients with negative serum AFP

Variables	Number (percentage)/Median (range)			*P* value
	Total patients	Training cohort	Validation cohort	
	(n = 509)	(n = 339)	(n = 170)	
Age, y	55 (23‐83)	55 (23‐83)	56 (24‐77)	.335
Sex (Female/Male)	49 (9.6%)/460 (90.4%)	36 (10.6%)/303 (89.4%)	13 (7.6%)/157 (92.4%)	.284
HBsAg (Negative/Positive)	55 (10.8%)/454 (89.2%)	37 (10.9%)/302 (89.1%)	18 (10.6%)/152 (89.4%)	.911
TB, μmol/L	13.6 (1.6‐259.2)	12.9 (1.6‐259.2)	14.7 (3.1‐119.1)	.074
ALB, g/L	42.2 (13.8‐53.4)	42.0 (13.8‐53.4)	42.5 (28.3‐50.5)	.295
ALT, IU/L	36.3 (6.9‐1156.1)	35.3 (6.9‐619.8)	39.1 (10.6‐1156.1)	.155
AST, IU/L	30.3 (9.9‐1304.3)	30.0 (9.9‐1009.9)	32.0 (11.8‐1304.3)	.276
GGT, IU/L	52.0 (8.0‐1767.0)	52.0 (8.0‐1767.0)	55.0 (8.0‐1391.0)	.506
LDH, IU/L	179.0 (27.0‐1077.0)	176.0 (27.0‐1077.0)	183.5 (87.0‐1042.0)	.123
ALP, U/L	82.0 (27.0‐429.0)	82.0 (27.0‐429.0)	82.5 (33.0‐408.0)	.657
CEA, μg/L	2.4 (0.2‐33.6)	2.3 (0.2‐15.2)	2.8 (0.4‐33.6)	***.028***
CA19‐9, μg/L	16.7 (0.6‐372.9)	16.1 (0.6‐372.9)	18.6 (0.6‐235.0)	.073
Tumor size, cm	4.0 (0.8‐20.7)	4.1 (1.0‐20.7)	4.0 (0.8‐20.0)	.298
Blood loss, mL (<400/≥400)	401 (78.8%)/108 (21.2%)	268 (79.1%)/71 (20.9%)	133 (78.2%)/37 (21.8%)	.831
HBV‐DNA load, IU/mL (<2000/≥2000)	297 (58.3%)/212 (41.7%)	197 (58.1%)/142 (41.9%)	100 (58.8%)/70 (41.2%)	.878
Tumor number (Single/Multiple)	468 (91.9%)/41 (8.1%)	311 (91.7%)/28 (8.3%)	157 (92.4%)/13 (7.6%)	.811
Major resection (No/Yes)	403 (79.2%)/106 (20.8%)	264 (77.9%)/75 (22.1%)	139 (81.8%)/31 (18.2%)	.308
MVI (No/Yes)	398 (78.2%)/111 (21.8%)	266 (78.5%)/73 (21.5%)	132 (77.6%)/38 (22.4%)	.833
Satellite lesions (No/Yes)	416 (81.7%)/93 (18.3%)	277 (81.7%)/62 (18.3%)	139 (81.8%)/31 (18.2%)	.988
Liver cirrhosis (No/Yes)	239 (47.0%)/270 (53.0%)	168 (49.6%)/171 (50.4%)	71 (41.8%)/99 (58.2%)	.097
Edmondson‐Steiner grade (I + II/III + IV)	154 (30.3%)/355 (69.7%)	103 (30.4%)/236 (69.6%)	51 (30.0%)/119 (70.0%)	.929
Child‐Pugh (A/B)	499 (98.0%)/10 (2.0%)	332 (97.9%)/7 (2.1%)	167 (98.2%)/3 (1.8%)	1.000
AJCC eighth (IA/IB/II/IIIA)	54 (10.6%)/328 (64.4%)/113 (22.2%)/14 (2.8%)	35 (10.3%)/216 (63.7%)/76 (22.4%)/12 (3.5%)	19 (11.2%)/112 (65.9%)/37 (21.8%)/2 (1.2%)	.417
BCLC stage(0/A/B)	54 (10.6%)/430 (84.5%)/25 (4.9%)	35 (10.3%)/284 (83.8%)/20 (5.9%)	19 (11.2%)/146 (85.9%)/5 (2.9%)	.341
JIS score (0/1/2)	35 (6.9%)/427 (83.9%)/47 (9.2%)	22 (6.5%)/284(83.8%)/33 (9.7%)	13 (7.6%)/143 (84.1%)/14 (8.2%)	.780

Abbreviations: AJCC, American Joint Committee on Cancer; ALB, albumin; ALP, alkaline phosphatase; ALT, alanine transaminase; AST, aspartate aminotransferase; BCLC, Barcelona Clinic Liver Cancer; CA19‐9, carbohydrate antigen 19‐9; CEA, carcino‐embryonic antigen; GGT, γ‐glutamyl transferase; HCC, hepatocellular carcinoma; HBsAg, hepatitis B surface antigen; HBV‐DNA, hepatitis B virus deoxyribonucleic acid; JIS, Japan Integrated Staging Score; LDH, lactate dehydrogenase; MVI, microvascular invasion; *P*: Training versus Validation Cohorts; TB, total bilirubin;

### 3.2 Patient prognosis

For training cohort patients, the 1‐, 2‐, 3‐, 4‐, and 5‐year OS rate and RFS rate were 91.4%, 83.2%, 80.4%, 72.7% and 67.1%; 79.9%, 69.5%, 61.7%, 55.0%, and 45.3%, respectively. Respective median RFS and follow‐up time were 56 and 60.2 months. For validation cohort patients, the 1‐, 2‐, 3‐, 4‐, and 5‐year OS rate and RFS rate were 95.3%, 88.7%, 81.2%, 76.4%, and 71.2%; 84.6%, 75.5%, 69.8%, 60.3%, and 50.7%, respectively. The median RFS time and follow‐up time were 64 and 60.1 months, respectively.

### 3.3 Identification of predictors of patient survival

Univariate and multivariate analysis of OS and RFS in the training cohort were conducted and were shown in Table 2. Multivariate analysis demonstrated that six factors were independent predictors of OS and RFS risk including alkaline phosphatase (ALP) (OS:HR = 1.005, 95%CI:1.001‐1.009, *P* = .025; RFS: HR = 1.004, 95%CI: 1.001‐1.008, *P* = .017), liver cirrhosis (OS: HR = 1.888, 95%CI:1.224‐2.914, *P* = .004; RFS: HR = 1.479, 95%CI:1.075‐2.036, *P* = .016), tumor size (OS: HR = 1.178, 95%CI:1.086‐1.278, *P* < .001; RFS: HR = 1.133, 95%CI:1.056‐1.216, *P* = .001), satellite lesions (OS: HR = 2.264, 95%CI:1.410‐3.637, *P* = .001; RFS: HR = 1.522, 95%CI:1.033‐2.242, *P* = .034), MVI (OS: HR = 1.688, 95%CI:1.057‐2.697, *P* = .029; RFS: HR = 1.461, 95%CI:1.014‐2.105, *P* = .042), Edmondson‐Steiner grade (OS: HR = 2.368, 95%CI:1.415‐3.962, *P* = .001; RFS: HR = 1.545, 95%CI:1.085‐2.202, *P* = .016). In addition, sex (HR = 2.231, 95%CI:1.205‐4.132, *P* = .011) and tumor number (HR = 1.757, 95%CI:1.114‐2.773, *P* = .015) were also independent risk factors for RFS.

**Table 2:** Univariate and multivariate analysis of OS and RFS in the training cohort

Variables	OS			RFS		
	HR	95%CI	*P* value	HR	95%CI	*P* value
Univariate analysis						
Age, y	0.995	0.977‐1.014	.608	0.995	0.981‐1.009	.483
Sex (Male, Female)	1.635	0.759‐3.522	.209	1.841	1.024‐3.310	***.042***
HBsAg (Negative/Positive)	0.871	0.487‐1.558	.641	1.177	0.722‐1.919	.513
TB, μmol/L	1.014	1.003‐1.024	***.010***	1.009	0.999‐1.020	.090
ALB, g/L	0.939	0.907‐0.972	***<.001***	0.952	0.925‐0.980	***.001***
ALT, IU/L	1.002	1.000‐1.005	.081	1.001	0.999‐1.004	.417
AST, IU/L	1.001	0.998‐1.003	.602	1.000	0.997‐1.002	.680
GGT, IU/L	1.001	1.001‐1.002	***.001***	1.001	1.000‐1.002	***.026***
LDH, IU/L	1.001	0.999‐1.003	.339	1.000	0.998‐1.002	.697
ALP, U/L	1.008	1.005‐1.010	***<.001***	1.005	1.002‐1.007	***<.001***
CEA, μg/L	1.007	0.903‐1.122	.904	1.010	0.929‐1.098	.815
CA19‐9, μg/L	1.005	1.001‐1.008	***.008***	1.002	0.999‐1.006	.227
Tumor size, cm	1.145	1.091‐1.203	***<.001***	1.099	1.053‐1.147	***<.001***
Blood loss, mL (<400/≥400)	1.796	1.179‐2.734	***.006***	1.460	1.037‐2.055	***.030***
HBV‐DNA load, IU/mL (<2000/≥2000)	1.498	1.019‐2.201	***.040***	1.172	0.869‐1.581	.299
Tumor number (Single/Multiple)	1.567	0.858‐2.861	.143	2.466	1.600‐3.799	***<.001***
Major resection (No/Yes)	1.943	1.285‐2.938	***.002***	1.531	1.093‐2.144	***.013***
MVI (No/Yes)	2.241	1.486‐3.378	***<.001***	1.860	1.333‐2.594	***<.001***
Satellite lesions (No/Yes)	2.685	1.775‐4.061	***<.001***	1.879	1.327‐2.660	***<.001***
Liver cirrhosis (No/Yes)	1.516	1.026‐2.240	***.037***	1.361	1.010‐1.835	***.043***
Edmondson‐Steiner grade (I + II/III + IV)	2.312	1.405‐3.805	***.001***	1.556	1.108‐2.184	***.011***
Child‐Pugh (A/B)	2.055	0.756‐5.586	.158	0.815	0.260‐2.553	.726
Multivariate analysis						
ALP, U/L	1.005	1.001‐1.009	***.025***	1.004	1.001‐1.008	***.017***
Liver cirrhosis (No/Yes)	1.888	1.224‐2.914	***.004***	1.479	1.075‐2.036	***.016***
Tumor size, cm	1.178	1.086‐1.278	***<.001***	1.133	1.056‐1.216	***.001***
Satellite lesions (No/Yes)	2.264	1.410‐3.637	***.001***	1.522	1.033‐2.242	***.034***
MVI (No/Yes)	1.688	1.057‐2.697	***.029***	1.461	1.014‐2.105	***.042***
Edmondson‐Steiner grade (I + II/III + IV)	2.368	1.415‐3.962	***.001***	1.545	1.085‐2.202	***.016***
Sex (Male, Female)				2.231	1.205‐4.132	***.011***
Tumor number (Single/Multiple)				1.757	1.114‐2.773	***.015***

Abbreviations: ALB, albumin; ALP, alkaline phosphatase; ALT, alanine transaminase; AST, aspartate aminotransferase; CA19‐9, carbohydrate antigen 19‐9; CEA, carcino‐embryonic antigen; GGT, γ‐glutamyl transferase; HBsAg, hepatitis B surface antigen; HBV‐DNA, hepatitis B virus deoxyribonucleic acid; LDH, lactate dehydrogenase; MVI, microvascular invasion; OS, overall survival; RFS, recurrence free survival; TB, total bilirubin.

*P* < .05 was defined as statistical significance.

### 3.4 OS and RFS nomogram construction and validation

**Figure 1:**
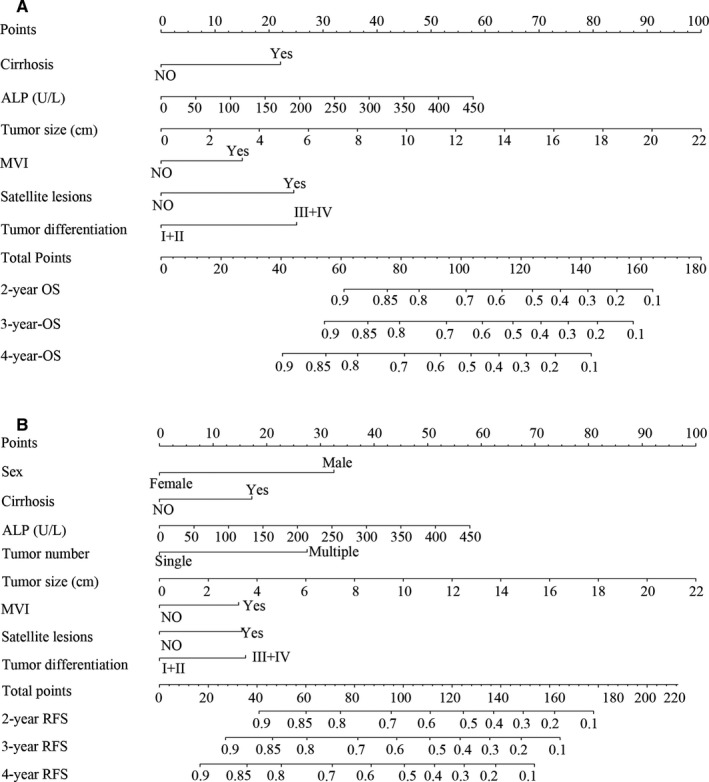
Nomograms for predicting prognosis in patients with AFP‐negative HCC. A, Overall survival (OS). B, Recurrence free survival (RFS). ALP, Alkaline phosphatase; MVI, microvascular invasion

**Figure 2:**
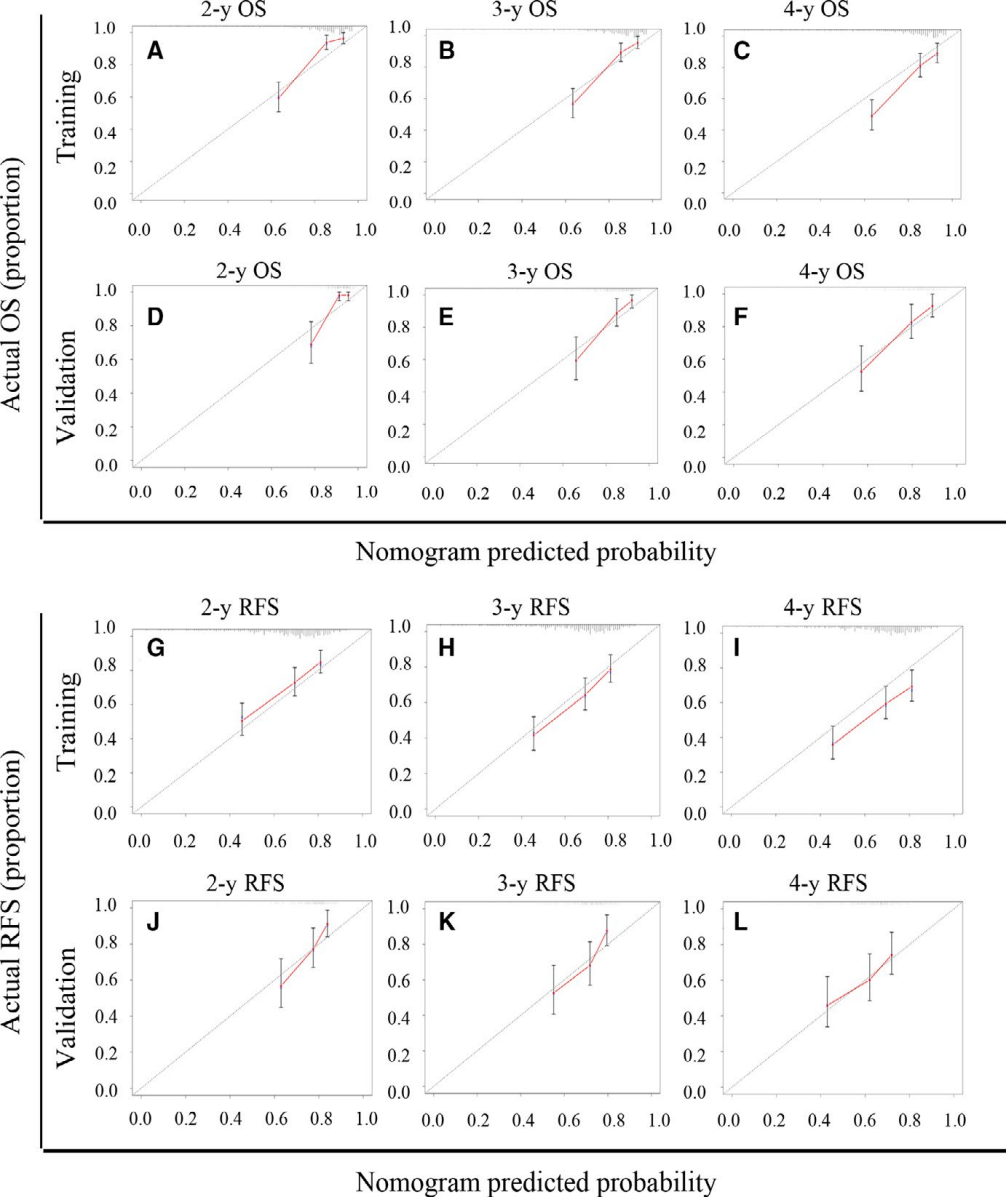
Calibration curves of nomograms for predicting overall survival (OS) and recurrence free survival (RFS) at 2‐,3‐and 4‐y. A‐C, 2‐,3‐and 4‐y OS in training cohort patients. D‐F, 2‐,3‐and 4‐y OS in validation cohort patients. G‐I, 2‐,3‐and 4‐y RFS in training cohort patients. J‐L, 2‐,3‐and 4‐y RFS in validation cohort patients

Those factors found to be independently predictive of patient survival outcomes in the multivariate analyses were performed to construct the nomograms. With respect to the nomogram of OS (Figure 1A), the training and validation cohort C‐index values were 0.742 (95%CI: 0.684‐0.800) and 0.740 (95%CI: 0.653‐0.827), respectively. As for the nomogram of RFS (Figure 1B), the training and validation C‐index values were 0.669 (95%CI: 0.623‐0.715) and 0.676 (95%CI: 0.606‐0.745), respectively. Moreover the calibration curves indicated that a good consistency between observed actual outcomes and predicted values for 2‐, 3‐, and 4‐year OS (Figure 2A‐F) and RFS (Figure 2G‐L) both in the two cohorts.

### 3.5 Comparison of the performance between nomograms and other prognostic models

Other prognostic models including AJCC eighth,^9^ BCLC staging system,^13^ JIS score^10^ and prognostic model for RFS constructed by Gan et al^17^were compared with the established nomogram in this study to identify which prognostic model had the more accurate ability of prediction (Table 3). The OS C‐index value in the training cohort was 0.742, and this was markedly increased relative to the AJCC eighth (0.632, 95%CI:0.583‐0.680, *P* < .001), BCLC stage (0.553, 95%CI:0.515‐0.591, *P* < .001) and JIS score (0.557, 95%CI: 0.519‐0.595, *P* < .001). Similarly, the OS C‐index value in the validation cohort was 0.740, and this was also increased relative to value for the AJCC eighth (0.576, 95%CI:0.503‐0.648, *P* < .001), BCLC stage (0.553, 95%CI:0.479‐0.587, *P* < .001) and JIS score (0.553, 95%CI: 0.497‐0.610, *P* < .001). The RFS C‐index value in the training cohort was 0.669, and was significantly higher than the AJCC eighth (0.608, 95%CI:0.570‐0.647, *P* < .001), BCLC stage (0.554, 95%CI:0.524‐0.584, *P* < .001), JIS score (0.551, 95%CI: 0.521‐0.581, *P* < .001), and model of Gan et al (0.636, 95%CI:0.591‐0.682, *P* = .012). In the validation cohort, the C‐index of the nomogram for RFS was 0.676, which was also significantly higher than the AJCC eighth (0.571, 95%CI:0.514‐0.629, *P* < .001), BCLC stage (0.529, 95%CI:0.486‐0.571, *P* < .001), JIS score (0.522, 95%CI: 0.478‐0.567, *P* < .001) and model of Gan et al (0.621, 95%CI:0.553‐0.690, *P* = .031). These results indicated that the established nomograms had the more accurate ability of predication than other models.

**Table 3:** C‐index of prognostic staging systems for OS and RFS

Prognostic system	Training cohort						Validation cohort					
	OS			RFS			OS			RFS		
	C‐index	95%CI	*P* value	C‐index	95%CI	*P* value	C‐index	95%CI	*P* value	C‐index	95%CI	*P* value
Nomogram	0.742	0.684‐0.800		0.669	0.623‐0.715		0.740	0.653‐0.827		0.676	0.606‐0.745	
AJCC eighth	0.632	0.583‐0.680	<.001	0.608	0.570‐0.647	<.001	0.576	0.503‐0.648	<.001	0.571	0.514‐0.629	<.001
BCLC stage	0.553	0.515‐0.591	<.001	0.554	0.524‐0.584	<.001	0.533	0.479‐0.587	<.001	0.529	0.486‐0.571	<.001
JIS score	0.557	0.519‐0.595	<.001	0.551	0.521‐0.581	<.001	0.553	0.497‐0.610	<.001	0.522	0.478‐0.567	<.001
Model of Gan et al				0.636	0.591‐0.682	.012				0.621	0.553‐0.690	.031

### 3.6 Assessment of the discriminative ability of nomograms

**Figure 3:**
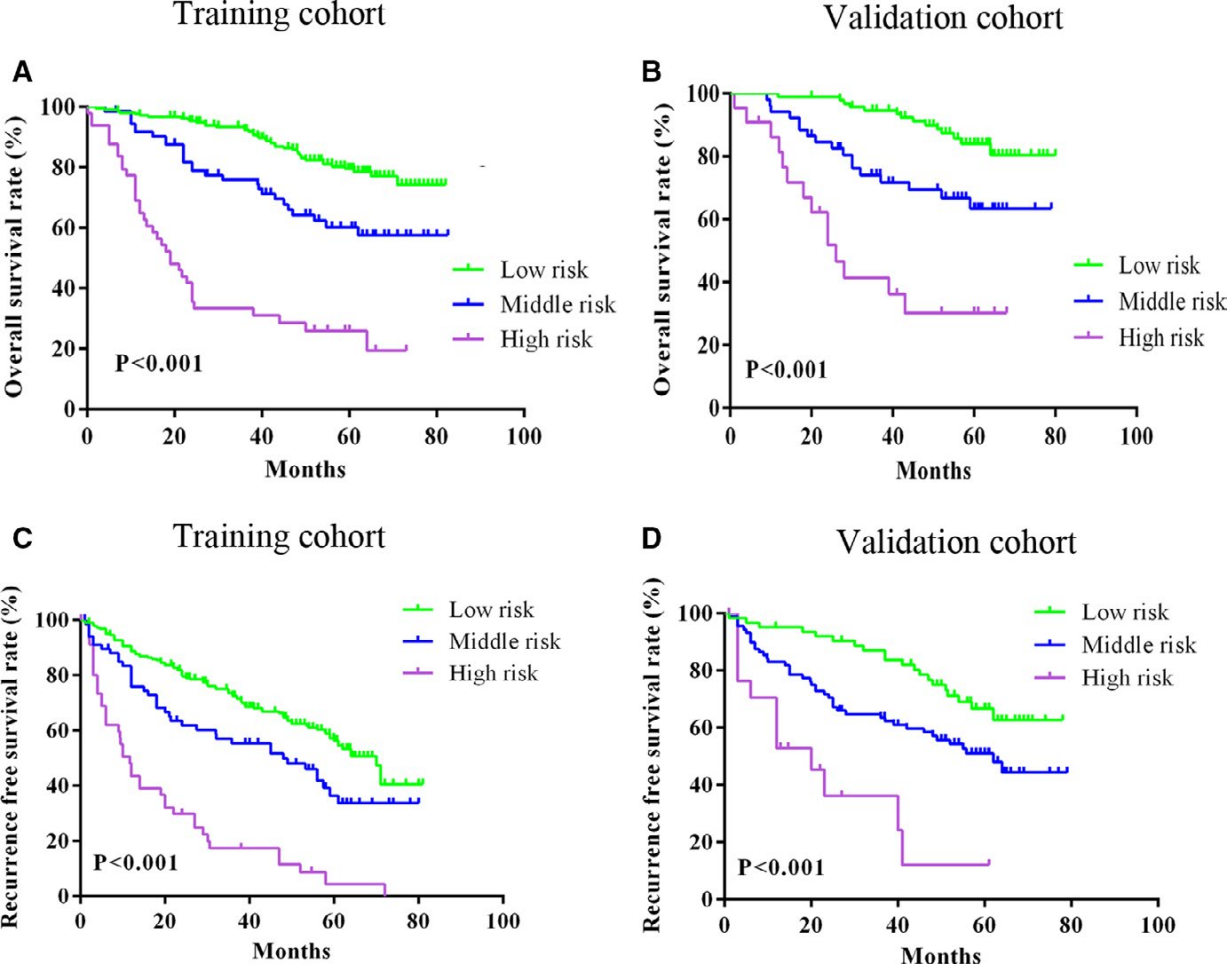
Kaplan‐Meier curves of risk groups according to points generated form nomograms. A‐B, Overall survival (OS) risk groups in the training and validation cohorts. C‐D, Recurrence free survival (RFS) risk groups in training and validation cohorts

The total points of each patient were generated from the established nomogram. With regard for OS, the total points in the training and validation cohorts were approximately ranged from 17.5 to 170 and 14 to 175, respectively. All patients were separated into low, middle and high risk groups by the cut‐off scores of 77.5 and 102.5 for the training cohort and 72.5 and 102.5 for the validation cohort. As depicted in Figure 3A‐B, the Kaplan‐Meier curves showed clean and distinct prognostic rate of OS in each risk group (*P* < .001). Similarly, all patients were divided into three risk groups based on cut‐off scores of 100 and 121.5 for the training cohort and 82.5 and 135 for the validation cohort, and distinct rate of RFS in each risk group also observed (*P* < .001) (Figure 3C‐D).

**Figure 4:**
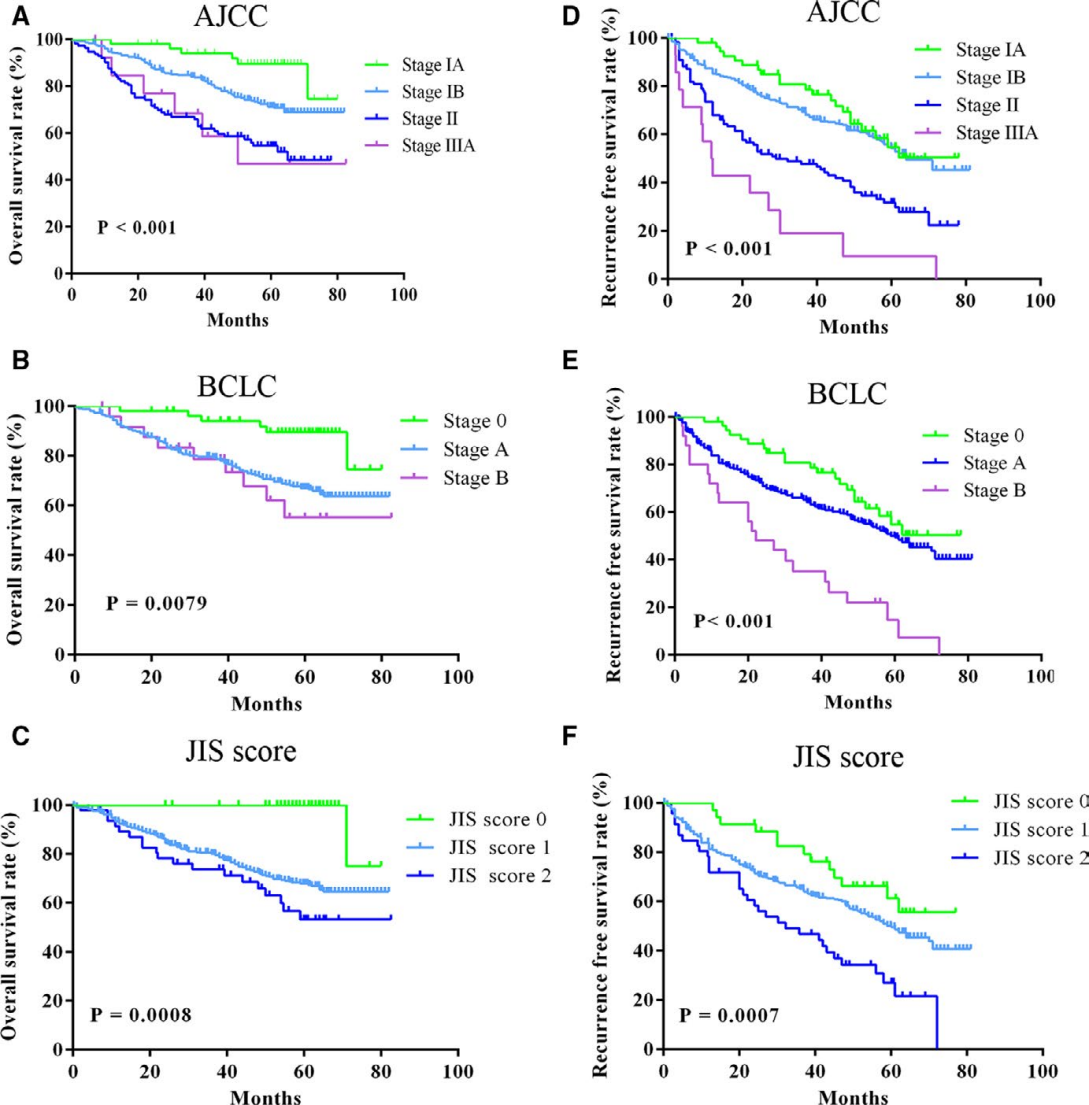
Kaplan‐Meier curves of overall survival (OS) and recurrence free survival (RFS) for conventional staging systems. A‐C, Kaplan‐Meier curves of OS for AJCC eighth edition (A), BCLC (B) and JIS score (C). D‐F, Kaplan‐Meier curves of RFS for AJCC eighth edition (D), BCLC stage system (E) and JIS score (F)

OS and RFS Kaplan‐Meier curves for conventional staging systems including AJCC eighth, BCLC stage and JIS score in all patients were depicted (Figure 4A‐F), respectively. These Kaplan‐Meier curves exhibited a distinct different prognostic stratum for each staging system in OS and RFS (*P* < .05 in all systems), however, overlapping curves were observed in almost all staging systems. On the contrary, distinct and non‐overlapping curves of OS and RFS were generated by the established nomograms, which indicated that the nomograms had a better discrimination ability than conventional staging systems.

### 3.7 Clinical application of the nomogram

**Figure 5:**
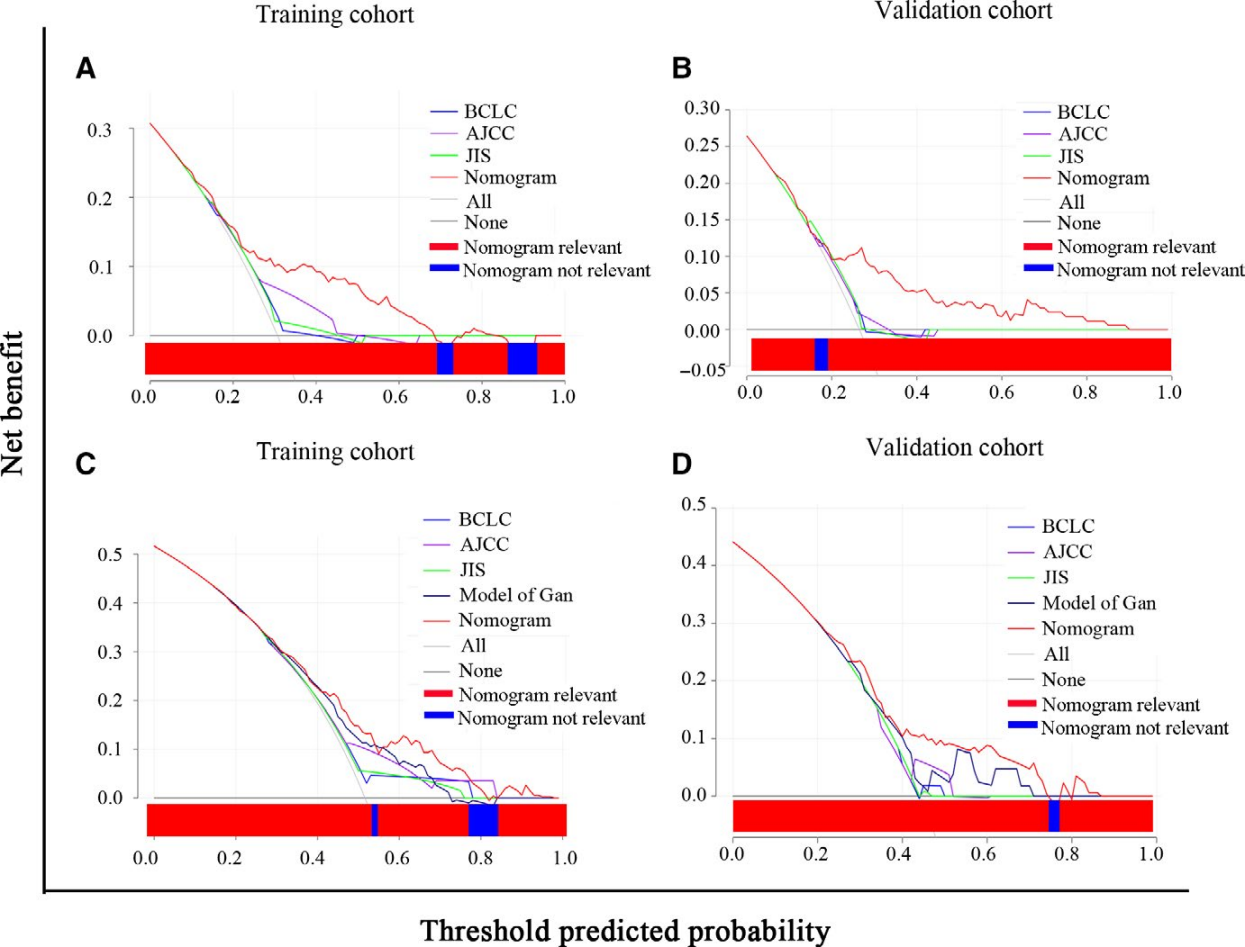
Decision curve analysis (DCA) for overall survival (OS) and recurrence free survival (RFS) of prognostic models. A‐B. DCA for OS in training (A) and validation (B) cohorts. C‐D, DCA for RFS in training (C) and validation (D) cohorts. The *x*‐axis and the *y*‐axis represent threshold probability and net benefit, respectively. The black line corresponds to no patients experiencing the indicated event, and the gray line corresponds to the death of all patients

DCA based on the net benefit and threshold probabilities was performed to assess the clinical value of these nomograms. As for OS, the nomogram demonstrated superior net benefit with a wide range of threshold probabilities relative to AJCC eighth, BCLC stage and JIS score models in the training and validation cohorts (Figure 5A‐B). Meanwhile, the nomogram also displayed a superior net benefit and improved performance than AJCC eighth, BCLC stage, JIS score and Gan et al models for RFS in the training and validation cohorts (Figure 5C‐D). The DCA curves indicated that the nomograms had superior clinical usefulness than other prognostic models.

## 4 DISCUSSION

In this study, a total of 509 AFP‐negative HCC patients were analyzed retrospectively following radical resection. Nomograms that were reasonably effective in predicting prognosis for OS and RFS based on independent risk factors were derived and validated. Our monograms shown more accurate predication when compared with other models, with C‐index values of 0.742 and 0.669 for OS and RFS in the training cohort, respectively, and 0.740 and 0.676 in the validation cohort, respectively. Moreover, a more satisfactory discrimination capability was also observed in the established nomograms for OS and RFS using Kaplan‐Meier curves. In addition, DCA demonstrated that these novel nomograms displayed a better net benefit and had superior clinical utility than other staging systems.

Several staging systems have been widely used for the treatment of HCC, especially the AJCC eighth TNM stage and BCLC stage. The eighth TNM stage stratifies HCC patients according to tumor status, lymph node stage and distant metastases, and could serve as treatment guidelines for HCC.^18^ However, TNM stage only considers tumor burden without other factors that affect tumor prognosis, which could result in bias in the treatment and prognosis of HCC.^27^ Thus, the applicability of TNM stage in clinical treatment is limited. The BCLC staging system consists of tumor stage, liver function, performance status, and cancer‐related symptoms, and is the most frequently used tool and complete evaluation system for prognostic stratification.^13^Furthermore, it is the only staging system able to provide therapeutic suggestions for each specified stage of HCC. A previous study reported that BCLC could significantly stratify and discriminate survival rate in HCC patients with AFP negative.^28^ The JIS score system composes of Japanese TNM stage and Child‐Pugh classification, and has been validated in multiple publications.^29^, ^30^ It is among the most widely used standard classification systems used in the Asia‐Pacific region. However, it has been revealed that BCLC stage and JIS score were limited to the stratification of advanced stage HCC patients.^31^, ^32^ Furthermore, the BCLC staging system was poor in distinguishing patients between stage A and B (*P* = .509) for OS, and stage 0 and A for RFS (*P* = .134), and JIS score was poor for differentiating patients between JIS scores 1 and 2 for OS (*P* = .132) and JIS scores 0 and 1 for RFS (*P* = .125) in the present study.

Fortunately, nomograms with more accurate prognostic prediction and superior stratify ability than traditional staging systems were developed and validated in several cancer types.^14^, ^15^ In the present study, nomograms comprising liver function, tumor status and clinicopathologic characteristics for OS and RFS in AFP‐negative HCC patients were constructed. Compared with the AJCC eighth TNM stage, our established nomograms included other factors affecting prognosis, which were not contained in TNM stage, exhibited a significantly higher prediction capability for OS and RFS according to the C‐indexes of training and validation cohorts. When compared with the BCLC stage and JIS score, our constructed nomograms also had a higher prediction accuracy for OS and RFS, with higher C‐index values in two cohorts (Table 3). This might be related to the inclusion of pathological features in our nomograms. The Kaplan‐Meier curves of OS and RFS for conventional staging systems in all patients were depicted, as shown in Figure 4. However, overlapping curves were observed in almost all staging systems. It means that the ability of these systems to predict patient survival was suboptimal. Luckily, our novel nomograms performed well in stratifying patients and discriminating survival outcomes in risk groups, which showed non‐overlapping and distinct Kaplan‐Meier curves compared with other systems (Figure 3). Moreover, DCA demonstrated that the nomograms for predicting OS and RFS were more beneficial than other staging systems in almost all ranges (Figure 5). Compared with two previous studies,^17^, ^18^ our nomograms also showed greater performance. However, two variables in the nomogram for OS constructed by Wang et al including BMI and distant metastases, which were not included in the present study. Thus, the comparison was unsatisfactory and not comparable. With respect to RFS, our nomogram contained all variables and demonstrated superior performance in prediction of recurrence compared with the model of Gan et al (*P* = .031). The DCA also demonstrated that the nomogram for predicting RFS was more beneficial than that of Gan et al’s model over almost complete range (Figure 5). This revealed that our nomograms are more accurate and powerful predictors of survival and recurrence in AFP‐negative HCC patients. In addition, surgeons can use the nomograms to develop personalized surveillance strategies for such patients and may be helpful for selection of patients for further therapy in clinical treatment.

In this study, independent risk factors such as ALP, liver cirrhosis, satellite lesions, MVI, tumor size, and Edmondson‐Steiner grade were associated with both OS and RFS, and sex and tumor number were additional risk factors for RFS. ALP is a hydrolytic enzyme widely found in the blood sinuses of liver cells and the bile duct membrane, related to the absorption and transport of certain substances. Increased serum ALP is associated with liver disease including HCC, cholangiocarcinoma, and biliary cirrhosis.^33^ High preoperative ALP was reportedly an independent risk factor for long‐time prognosis of HCC.^34^ Moreover high levels of ALP may increase the risk of death in patients with HCC after hepatectomy.^35^ Our nomograms for OS and RFS also showed that higher levels of ALP indicated worse prognosis for HCC. Interestingly, sex represented a large weighting in the nomogram for RFS and was significantly associated with HCC recurrence. Previous studies have found that men were more likely to develop HCC than women, and male patients were more prone to relapse than female patients in 2 years after hepatectomy.^36^, ^37^ This sex difference may be related to sex hormones and requires additional study.

The presence of MVI has been confirmed to be associated with intrahepatic metastasis, and the risk of MVI increasing with tumor size and tumor numbers.^38^ Literatures had showed that MVI was detected in 15.0%‐57.1% of HCC,^22^ and 60%‐90% of tumor sizes greater than 5 cm.^39^ In the present study, the presence of MVI was positive in 73 cases (21.5%) and 38 cases (22.4%) in both training and validation cohorts, respectively, which was consistent with the results reported in the literature. Satellite lesions were also linked with tumor invasion and metastasis. Previous studies demonstrated that MVI and satellite lesions were negative risk factors for tumor recurrence and long‐term survival, possibly related to the multicentric carcinogenic mechanism and intrahepatic metastasis of HCC.^40^, ^41^ In our nomograms, patients with MVI and satellite lesions had poor prognosis.

Tumor size was positively correlated with the tumor recurrence, especially for those with a diameter greater than 5 cm.^36^ This effect may be related to the fact that larger tumors more likely to result in intrahepatic metastasis and vascular invasion.^42^ However, tumor size has been found to not be directly related to prognosis of HCC in a number of reports.^43^ In the current study, we found that tumor size was an independent predictor for OS and RFS in AFP‐negative HCC patients. For tumor size scores up to 100 points, larger tumors resulted in worse prognosis. Moreover multiple lesions were linked significantly to HCC recurrence in the present study, possibly related to the increased aggressive behavior of the tumors.

Liver cirrhosis is a well‐recognized precancerous lesion. Almost 90% of HCC patients progressed form hepatitis B or C and liver cirrhosis.^44^ Multiple studies have demonstrated that liver cirrhosis was a negative risk factor for survival and postoperative multicenter recurrence.^45^, ^46^ In addition to liver cirrhosis, Edmondson‐Steiner grade was also an independent predictor of OS and RFS. It is worth noting that Edmondson‐Steiner grade was not contained in any of the widely used staging systems for HCC. However, liver cirrhosis and Edmondson‐Steiner grade were key factors in our nomograms and demonstrated negative prognosis.

The present study has multiple limitations. First, the nomograms were established using data from a single center data that were retrospective, and so the results need to be further validated via additional prospective studies. Second, the samples included in our cohorts were small. Multi‐center studies with a large sample size are required to test the nomograms performance. Third, the nomograms were only suitable for postoperative decision‐making rather than preoperative in AFP‐negative HCC patients.

## 5 CONCLUSIONS

In conclusion, simple but powerful nomograms using independent risk factors were developed and validated for predicting survival and recurrence in AFP‐negative HCC patients after radical resection. These novel nomograms displayed superior performance and discriminative power relative to conventional staging systems, suggesting they are of potential value for clinicians when guiding surgery, treatment, or monitoring strategies in patients.

## CONFLICTS OF INTEREST

The authors declare that they have no conflict of interest.

## AUTHOR CONTRIBUTIONS

Jian Huang and Fu‐Chen Liu were involved in the conception and design of the study; the acquisition, analysis, and interpretation of data; and drafted the manuscript. Li Li and Wei‐Ping Zhou were involved in the design of the study and analysis and interpretation of the data. Bei‐Ge Jiang and Ze‐Ya Pan were involved in the conception and design of the study; the acquisition, analysis, and interpretation of data; and drafted and revised the manuscript.

## Data Availability

Research data are not shared, owing to the privacy or ethical restrictions.
